# A polymorphic AT-repeat causes frequent allele dropout for an *MME* mutational hotspot exon

**DOI:** 10.1136/jmedgenet-2021-108281

**Published:** 2022-03-22

**Authors:** Helle Høyer, Hilde T Hilmarsen, Raute Sunder-Plassmann, Geir J Braathen, Peter M Andersen, Christian Beetz, Sandra Hacker, Øystein L Holla, Ingo Kurth, Wolfgang N Löscher, Simone B C F Reiter, Sabine Rudnik-Schöneborn, Linda Strand, Reinhard Windhager, Martina Witsch-Baumgartner, Jan Senderek, Michaela Auer-Grumbach

**Affiliations:** 1 Department of Medical Genetics, Telemark Hospital, Skien, Norway; 2 Department of Laboratory Medicine, Medical University of Vienna, Vienna, Austria; 3 Department of Clinical Sciences, Norrlands universitetssjukhus, Umeå, Sweden; 4 Centogene AG, Rostock, Germany; 5 Department of Orthopaedics and Trauma Surgery, Medical University of Vienna, Vienna, Austria; 6 Institute of Human Genetics, Medical Faculty, RWTH Aachen University, Aachen, Germany; 7 Department of Neurology, Medical University of Innsbruck, Innsbruck, Austria; 8 Department of Medical Genetics, Haukeland University Hospital, Bergen, Norway; 9 Institute of Human Genetics, Medical University of Innsbruck, Innsbruck, Austria; 10 Friedrich Baur Institute, Department of Neurology, Ludwig-Maximilians-Universität München Medizinische Fakultät, Munich, Germany

**Keywords:** allelic imbalance, DNA repeat expansion, neuromuscular diseases, sequence analysis, DNA, human genetics

Pathogenic variants in the *MME* gene cause dominant and recessive late-onset axonal hereditary neuropathy, that is, axonal Charcot-Marie-Tooth syndrome (LOCMT2). Here, we report next-generation sequencing (NGS) and Sanger sequencing (SS) results of 28 LOCMT2 patients carrying either the repeatedly reported c.467del p.(Pro156Leufs*14) or the c.440–2A>C variants. We demonstrate that an intronic AT-repeat in close proximity to these two mutations is frequently causing an allele dropout during SS that result in false genotyping in a considerable proportion of patients. This may result in an incorrect diagnosis, which has a considerable clinical impact for genetic counselling and prognosis.

Recent studies have demonstrated that both heterozygous and biallelic variants in *MME* (encoding the metalloprotease neprilysin) are a frequent cause of LOCMT2 (MIM: 617017).^1–3^ The heterozygotes variants cause a milder phenotype with reduced penetrance. Besides the large spectrum of rare or even single pathogenic *MME* variants, the frameshift deletion c.467del p.(Pro156Leufs*14) and the splice site mutation c.440–2A>C have been recurrently reported in patients with autosomal dominant and autosomal recessive LOCMT2.^2–4^ Although PCR is considered to be a robust technology and a reliable tool to be used for routine diagnosis, allele-specific sequence variations occasionally may provoke amplification failure of one of the two alleles at a given locus.^5^ Such an allele dropout has also been shown for the c.467del mutation in *MME* in one consanguineous family.^4^

In this study, registries at the Medical University of Vienna and Telemark Hospital Trust were searched for LOCMT2 individuals carrying the *MME* variants NM_007289.3:c.467del p.(Pro156Leufs*14) and NM_007289.3:c.440–2A>C. We ascertained 28 individuals from 16 families (MH1-MH16) afflicted with LOCMT2. For segregation analysis three healthy family members were also included. The families originated from Austria, Germany, Norway and Sweden.

Whole exome sequencing, NGS-based multigene panel sequencing or SS of the *MME* gene was performed and analysed as reported previously.^3^ SS was used to confirm *MME* variants detected by NGS and for segregation analysis in families. Due to conflicting results between NGS and SS at both laboratories, sequencing was repeatedly carried out using primers either including or excluding the adjacent AT-repeat of variable size, c.439+33_439+48AT[8-15], located 57 bp 5’ of exon 6 (online supplemental material 1). Subsequently, six additional enzymes and two additional conditions for the original AccuPrime Taq DNA Polymerase System were tested to unravel the PCR enzymes’ ability to amplify both the short and the long AT-repeat. A full list of the primers, enzymes and conditions used is described in the online supplemental material 1.

10.1136/jmedgenet-2021-108281.supp1 Supplementary data

Moreover, the length of the intronic AT-repeats was assessed on 179 selected DNA samples by using NGS data, fragment length analysis (FLA) and/or multiplex ligation-dependent probe amplification (MLPA). FLA details are described in the online supplemental material 1, MLPA and NGS followed procedures as described.^2 3^

Tracking of the c.467del p.(Pro156Leufs*14) and the c.440–2A>C *MME* mutations previously detected by NGS or SS revealed conflicting results in 7/28 (25%) of the patients when using the original SS primers including the AT-repeat (table 1). In three families, MH-1, MH-2 and MH-6, the c.467del variant was first detected as heterozygous by NGS, but turned out to be homozygous by SS in several family members. On the other hand, in family MH-14, the index patient was tested heterozygous for the c.440–2A>C variant by NGS, whereas the same mutation was absent by SS. To unravel these discrepancies, an alternative primer-set excluding the intronic AT-repeat was used for SS. This enabled a correct determination of the *MME* mutation status. The pedigrees and sequence traces are depicted in figure 1A, B. Additional sequences traces are provided in the online supplemental material 1. Furthermore, the length of the AT-repeat was tested by NGS and/or FLA and MLPA. Thereby, it turned out, that all seven patients with contradicting results were compound heterozygous for a short (8–9) and an expanded (13–15) AT-repeat, suggesting allele dropout of the expanded AT-allele. The incorrect determination of the mutation status did not occur in 17 patients who were homozygous for a short or a long AT-repeat on NGS/FLA (table 1).

**Table 1:** Summary of results from SS, NGS and FLA in families MH-1 to MH-16

Family ID	Patient ID	Result NGS	Result SS including AT-repeat	Result SS excluding AT-repeat	AT-repeat SS	AT-repeat NGS	AT-repeat FLA
MH-1	3	c.467del/WT	**c.467del/c.467del**	c.467del/WT	**8x/8x**	8x/13x	8x/13x
MH-1	6	c.467del/WT	**c.467del/c.467del**	c.467del/WT	**8x/8x**	8x/13x	ND
MH-1	7	c.467del/WT	**c.467del/c.467del**	c.467del/WT	**8x/8x**	8x/13x	8x/13x
MH-1	9	ND	c.467del/WT	c.467del/WT	8x/8x	ND	8x/8x
MH-1	8	WT/WT	WT/WT	WT/WT	13x/13x	13x/13x	13x/13x
MH-1	4	WT/WT	WT/WT	WT/WT	13x/13x	13x/13x	13x/13x
MH-2	5	c.467del/WT	c.467del/WT	c.467del/WT	8x/8x	8x/8x	8x/8x
MH-2	6	ND	**c.467del/c.467del**	c.467del/WT	**8x/8x**	ND	ND
MH-2	7	WT/WT	WT/WT	WT/WT	8x/8x	8x/8x	8x/8x
MH-3	–	c.467del/WT	c.467del/WT	ND	8x/8x	ND	ND
MH-3	–	ND	c.467del/WT	ND	8x/8x	ND	ND
MH-3	–	ND	c.467del/WT	ND	8x/8x	ND	ND
MH-4	–	c.467del/WT	c.467del/WT	c.467del/WT	8x/8x	8x/8x	8x/8x
MH-5	–	c.467del/WT	c.467del/WT	c.467del/WT	8x/8x	8x/8x	8x/8x
MH-6	4	c.467del/WT	c.467del/WT	c.467del/WT	8x/8x	8x/8x	8x/8x
MH-6	2	ND	c.467del/WT	c.467del/WT	8x/8x	ND	8x/8x
MH-6	7	ND	c.467del/WT	c.467del/WT	8x/8x	ND	8x/8x
MH-6	5	ND	c.467del/WT	c.467del/WT	8x/8x	ND	8x/8x
MH-6	9	ND	c.467del/WT	c.467del/WT	8x/8x	ND	8x/8x
MH-6	8	c.467del/WT	**c.467del/c.467del**	c.467del/WT	**8x/8x**	8x/13x	8x/15x
MH-6	10	c.467del/WT	**c.467del/c.467del**	c.467del/WT	**8x/8x**	8x/13x	8x/14x
MH-7	–	c.467del/c.467del	c.467del/c.467del	c.467del/c.467del	8x/8x	8x/8x	8x/8x
MH-8	–	c.467del/WT	c.467del/WT	c.467del/WT	8x/8x	8x/8x	8x/8x
MH-9	–	c.467del/WT	c.467del/WT	c.467del/WT	8x/8x	8x/8x	8x/8x
MH-10	–	c.467del/c.467del	c.467del/c.467del	c.467del/c.467del	8x/8x	8x/8x	8x/8x
MH-11	–	c.467del/WT	c.467del/WT	c.467del/WT	8x/8x	8x/8x	8x/8x
MH-12	–	c.440–2A>C/WT	c.440–2A>C/WT	c.440–2A>C/WT	13x/13x	ND	13x/13x
MH-13	–	c.440–2A>C/WT	c.440–2A>C/WT	c.440–2A>C/WT	13x/13x	13x/13x	13x/13x
MH-14	3	c.440–2A>C/WT	**WT/WT**	c.440–2A>C/WT	**8x/8x**	8x/13x	8x/13x
MH-15	–	c.440–2A>C/WT	c.440–2A>C/WT	–	13x/13x	13x/13x	13x/13x
MH-16	–	ND	c.440–2A>C/WT	ND	13x/13x*	ND	ND

Results of NGS, FLA and SS using different primers with and without the AT-repeat. Contradicting results are highlighted in bold.

Patient ID are listed according to the numbers on the pedigrees (figure 1A).

*Due to lack of DNA, complete testing was not possible, but a homozygous long allele can be concluded from results obtained in the control group (data not shown). Reference sequence according to NM_007289.3.

FLA, fragment length analysis; ND, no data/no DNA; NGS, next-generation sequencing; SS, Sanger sequencing; WT, wildtype.

**Figure 1:**
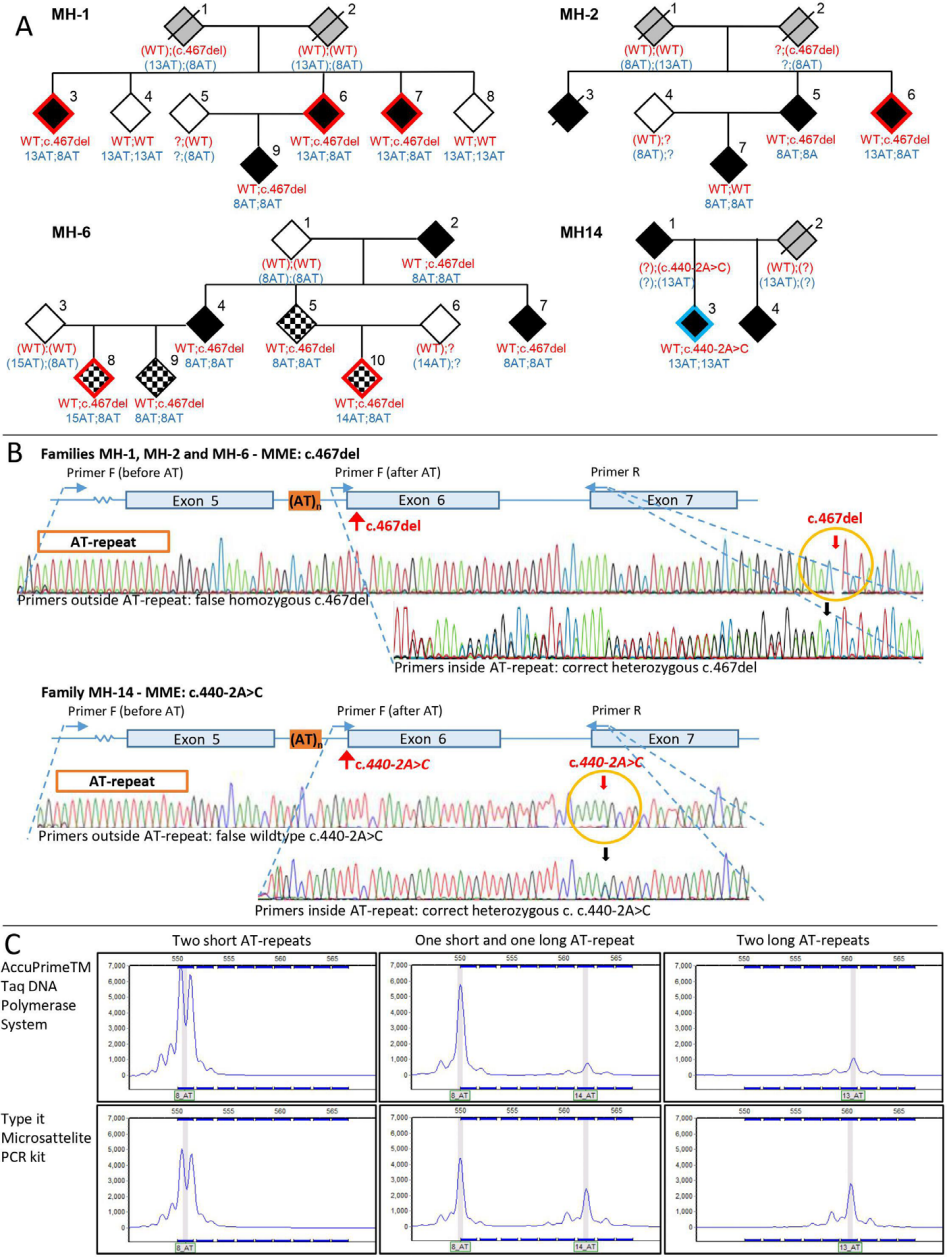
(A) Pedigrees of the families (MH-1, MH-2, MH-16, MH-14) with conflicting results. The alleles for the mutations c.467del and c.440–2A>C are shown in red. The alleles for the AT-repeat are shown in blue. Brackets indicate estimated alleles. Empty: unaffected individuals; black: affected individuals; chessboard filling: unaffected/asymptomatic mutation carriers. Symbols with red frame: individuals with false result (homozygous) by Sanger sequencing (SS). Symbol with blue frame: individual with false result (wildtype) by SS. (B) Outline of exon 5–7 (NM_007289.3) of the *MME* gene. Exons (blue boxes), AT-repeat (orange), *MME* mutations (red arrows) and primers situated 5’ and 3’ of the AT-repeat (blue arrows) are marked. Top: SS traces from individual false homozygous for the c.467del mutation (top sequence trace) obtained with forward primer situated 5’ of the AT-repeat and correct heterozygous c.467del result (lower sequence trace) obtained with forward primer situated 3’ the AT-repeat. The false homozygous c.476del mutation is indicated by a yellow circle and a red arrow, the correct (heterozygous) c.476del mutation is indicated by a black arrow. Bottom: SS traces from an individual with a false wildtype at position c.440–2A>C (top sequence trace) obtained with a forward primer situated 5’ of the AT-repeat and correct result (heterozygous, lower sequencing trace) obtained with a forward primer situated 3’ of the AT-repeat. False wildtype at position c.440–2A>C is indicated by a yellow circle and a red arrow, correct heterozygous c.440–2A>C is indicated by a black arrow. (C) Fragment length analysis with AccuPrime Taq DNA (top) and Type-it Microsatellite PCR kit (bottom) analysed for three individuals. The fluorescence signal from each *MME* AT-repeat is shown as blue peak in the chart and the length of the AT-repeat is indicated by grey boxes below the peak. Acceptable error limits of repeat sizing are ±1. The fluorescence signal intensity (y-axis) is set at 7000. Reference sequence according to NM_007289.3.

To determine the frequency of long and short AT-repeats, results of 179 additional individuals were evaluated. Thereby, 34% carried a homozygously short (up to 9 AT-repeats), 22% a homozygously long AT-repeat (12–16 AT-repeats) and 44% where compound heterozygous, giving an allele frequency of 56% for a short and 44% for a long AT-repeat. Data from the gnomAD database (broadinstitute.org) show a similar distribution. In the non-Finish European population, the allele frequency is 62.4% for a short and 37.6% for a long AT-repeat. The high frequency of a compound heterozygous AT-repeat bears a high risk to achieve incorrect results.

Finally, we investigated whether the use of different PCR enzymes circumvents allelic dropouts. These experiments showed that only three out of eight enzymes tested and none of the two specific conditions tested were able to amplify the expanded AT-allele in the presence of a short AT-allele (online supplemental material 1).

FLA indicated that the allele dropout likely occurs during PCR. Comparison of signal intensities for the AccuPrime enzyme and the Type-it Microsatellite PCR kit, of which the first caused allele dropout in SS whereas the latter not, showed that the signal intensity from the expanded AT-allele was substantially lower with the AccuPrime enzyme (figure 1C).

In summary, we identified that a short increase in an AT-repeat close to two *MME* hotspot mutations leads to allele dropout during SS and subsequent false interpretation of the results in several patients. A false positive prediction of a homozygous *MME* mutation would imply earlier onset, a more severe disease course and a high recurrence risk to offspring, whereas a false negative diagnosis could influence further diagnostic and therapeutic procedures and has an impact for genetic counselling. Although to date, NGS is frequently applied for routine diagnostics, SS is still used for verification of a particular variant and segregation analysis in a family. The fact that a small increase in a repetitive sequence may lead to amplification failure is important to bear in mind when designing primers for SS as it may be relevant for other genes as well.

## Data Availability

Data supporting the findings of this study are available from the corresponding author on reasonable request.

## References

[1] Higuchi Y , Hashiguchi A , Yuan J , Yoshimura A , Mitsui J , Ishiura H , Tanaka M , Ishihara S , Tanabe H , Nozuma S , Okamoto Y , Matsuura E , Ohkubo R , Inamizu S , Shiraishi W , Yamasaki R , Ohyagi Y , Kira J-ichi , Oya Y , Yabe H , Nishikawa N , Tobisawa S , Matsuda N , Masuda M , Kugimoto C , Fukushima K , Yano S , Yoshimura J , Doi K , Nakagawa M , Morishita S , Tsuji S , Takashima H . Mutations in MME cause an autosomal-recessive Charcot-Marie-Tooth disease type 2. Ann Neurol 2016;79:659–72. 10.1002/ana.24612 26991897 PMC5069600

[2] Auer-Grumbach M , Toegel S , Schabhüttl M , Weinmann D , Chiari C , Bennett DLH , Beetz C , Klein D , Andersen PM , Böhme I , Fink-Puches R , Gonzalez M , Harms MB , Motley W , Reilly MM , Renner W , Rudnik-Schöneborn S , Schlotter-Weigel B , Themistocleous AC , Weishaupt JH , Ludolph AC , Wieland T , Tao F , Abreu L , Windhager R , Zitzelsberger M , Strom TM , Walther T , Scherer SS , Züchner S , Martini R , Senderek J . Rare variants in MME, encoding metalloprotease neprilysin, are linked to late-onset autosomal-dominant axonal polyneuropathies. Am J Hum Genet 2016;99:607–23. 10.1016/j.ajhg.2016.07.008 27588448 PMC5011077

[3] Senderek J , Lassuthova P , Kabzińska D , Abreu L , Baets J , Beetz C , Braathen GJ , Brenner D , Dalton J , Dankwa L , Deconinck T , De Jonghe P , Dräger B , Eggermann K , Ellis M , Fischer C , Stojkovic T , Herrmann DN , Horvath R , Høyer H , Iglseder S , Kennerson M , Kinslechner K , Kohler JN , Kurth I , Laing NG , Lamont PJ , Wolfgang N L , Ludolph A , Marques W , Nicholson G , Ong R , Petri S , Ravenscroft G , Rebelo A , Ricci G , Rudnik-Schöneborn S , Schirmacher A , Schlotter-Weigel B , Schoels L , Schüle R , Synofzik M , Francou B , Strom TM , Wagner J , Walk D , Wanschitz J , Weinmann D , Weishaupt J , Wiessner M , Windhager R , Young P , Züchner S , Toegel S , Seeman P , Kochański A , Auer-Grumbach M . The genetic landscape of axonal neuropathies in the middle-aged and elderly: Focus on MME. Neurology 2020;95:e3163–79. 10.1212/WNL.0000000000011132 33144514 PMC7836667

[4] Dupuis M , Raymackers JM , Ackermans N , Boulanger S , Verellen-Dumoulin C . Hereditary axonal neuropathy related to MME gene mutation in a family with fetomaternal alloimmune glomerulonephritis. Acta Neurol Belg 2020;120:149–54. 10.1007/s13760-020-01275-9 31974930

[5] Blais J , Lavoie SB , Giroux S , Bussières J , Lindsay C , Dionne J , Laroche M , Giguère Y , Rousseau F . Risk of misdiagnosis due to allele dropout and false-positive PCR artifacts in molecular diagnostics. J Mol Diagn 2015;17:505–14. 10.1016/j.jmoldx.2015.04.004 26146130

