# Supplementary material for: A polymorphic AT-repeat causes frequent allele dropout for an MME mutational hotspot exon
Source: J Med Genet. 2022 Mar 22;59(10):1024–6. doi: 10.1136/jmedgenet-2021-108281 (PMC9554250; doi:10.1136/jmedgenet-2021-108281)

Supplementary figure and tables

MME reference sequence and primers.

Reference sequence and numbering is according to *MME* ENSG00000196549. Exon numbering and variant position is according to *MME* NM_007289.3. Exons are indicated in light blue, AT-repeat, c.439+33_439+48AT[8-15], in orange and the two *MME* mutations c.440-2A>C and c.467del in red. Primers used are indicated with black arrows and primer numbering.

E5F

92661 CATCACTGTTCATAAAAAAAAAAAAAAAGATAATCTAGTCAAAAGGACTTTCATCTAAATTGGCTGATTAAACTGACTCCCAGTTCTGTGCTAGGAATGAT

F1

92761 AATCTGGTAAGGGGTTTGGTTTTTCCTATAATGTTTTGGAACTGGGGGGAGGAAATGAATGTACCTCCAGAAAAGCAAGGTTTTTTTTTAACTGAACCCA

92861 CTTTGCAAATGTTAATTACTGCAAATGAGCAATTATGTTTGCATAGTGCAAATGAGCAATTATGTTGATGCATTTTATTAAATGTCCTATTTCAG**ATGTC**

Exon 5

92961 **CTTCAAGAACCCAAAACTGAAGATATAGTAGCAGTGCAGAAAGCAAAAGCATTGTACAGGTCTTGTATAAATGAAT**GTAAGTGCTCTTCATTTGATTTCA

AT-repeat F2 *MME*: c.440-2A>C

93061 TTAGGAGT**ATATATATATATATATAT**TGGTGCCAAACTATGCCTAGATTTCTAATTGAATTTATGTTTGTTGTTTCCAAAAG**CTGCTATTGATAGCAGAGGT**

MME: c.467del Exon 6

93161 **GGAGAACCTCTACTCAAACTGTTACCAGACATATATGGGTGGCCAGTAGCAACAGAAAACTGGGAGCAAAAATATG**GTAAGGCAATTTTCCTACTAAAAA

R2 R1

93261 AGAAATTTCCATGTAAAATCTATGTTATAATATCATTAGTGAACTAAAGCTTCTAAAGATATATTTTATCTTTTATTGCTTTAG**GTGCTTCTTGGACAGC**

Exon 7

93361 **TGAAAAAGCTATTGCACAACTGAATTCTAAATATGGGAAAAAAGTCCTTATTAATTTGTTTGTTGGCACTGATGATAAGAATTCTGTGAATCATGTAATT**

93461 **CAT**GTAAGTTTGTGTGTCAAATAACTAAAGTTACCTTTAAATTGTAATAAAACTTCCACATACATTGTTGTTATTGATATTTAGAAATAATGCTATTTTC

E7R

93561 AATTGAATTGAATAAGCAAGGAGGTAGTATATCCATGCTCTGTCCCTAATACCTGGAGTTCCAATTTTGAAAGTATTTTCTTAATGCTGTTTTCGACATT

93661 CACCCCTTGCTTCTGCTACCTCTGTTTTAGATACCTCTCCCTTCACAAAACAAATGATAAGAAGGGCTCCAGGCCCCTCCTATTCAGAAAAAGTTCCTAA

Primer name	Direction	Primer sequence	
Primers situated outside the AT-repeat			
F1	5’	GGGAGGAAATGAATGTACCTCCAGA	Standard primers at Telemark Hospital Trust
R1	3’	GCTGTCCAAGAAGCACCTAAAGCA	
E5F	5’	ATAATCTGGTAAGGGGTTTGGT	Standard primers at the Medical University of Vienna. For more information see also Auer-Grumbach et al., The American Journal of Human Genetics 2016, 99:607–623
E7R	3’	TGTTTTCGACATTCACCCC	
Primer situated inside the AT-repeat			
F2	5,	TCCAAAAGCTGCTATTGATAGC	
R2	3’	CCAAGAAGCACCTAAAGCAATA	

Enzymes and conditions

Standard Sanger sequencing conditions at Telemark Hospital Trust (top) and the Medical University of Vienna (bottom)

PCR enzyme	PCR primers	PCR reagents	PCR conditions	Comment	Result
Accuprime (ThermoFischer Scientific)	F1+R1	10x AccuPrime buffer II 1.25µl<br>Primer F 2µM 1,13µl<br>Primer R 2µM 1,13µl<br>Accuprime 2U/ul 0,25µl<br>dH_2O 7.75µl<br>DNA ≈50ng/ul 1.00µl	95ºC 2min + 95ºC 30s, 61ºC 30s , 72ºC 1min x 35 cycles + 72ºC 10 min + 4ºC →	Standard conditions and Telemark Hospital Trust. PCR clean-up was performed with ExoSAP IT Express, sequencing with BigDye v.3.1 Terminator Cycle Sequencing Kit and sequencing clean-up with BigDye XTerminator Purification Kit (all ThermoFicher Scientific). The products were sequenced on the ABI3130XL (Life Technologies Ltd., Paisley, UK and analysed with CLC Main Workbench (CLC bio, Aarhus, Denmark).	Allele drop-out of 13AT allele
Phusion Green Hot Start II High-Fidelity DNA Polymerase (Thermo Scientific)	E5F+E7R	2x Phusion Green MM 10.00µl<br>Primer F 1µM 0.50µl<br>Primer R 1µM 0.50µl<br>Nuclease free dH_2O 8.00µl<br>DNA ≈50ng/ul 1.00µl	98ºC 30s + 98ºC 5s, 63ºC 15s, 72ºC 15s x 30 cycles + 72ºC 5min + 4ºC →	Standard conditions at the Medical University of Vienna<br>PCR clean-up was performed with ExoSAP IT Express, sequencing with BigDye v.3.1 Terminator Cycle Sequencing Kit and sequencing clean-up with BigDye XTerminator Purification Kit (all Thermo Scientific). The products were sequenced on the ABI3130XL and analysed with Seqscape (Life Technologies Ltd., Paisley, UK)	Allele drop-out of 13AT allele

Ability of different PCR enzymes and conditions to amplify both the short and the long AT-repeat during Sanger sequencing.
The testing was performed on one individual homozygous for an 8AT repeat, one homozygous for a 13AT repeat and one heterozygous for an 8AT/13AT repeat. The test was repeated once for all individuals and additionally for four individuals heterozygous for an 8AT/13AT repeat.

Sanger sequencing conditions were similar for all enzyme and conditions tested. PCR clean-up was performed with ExoSAP IT Express, sequencing with BigDye v.3.1 Terminator Cycle Sequencing Kit and sequencing clean-up with BigDye XTerminator Purification Kit (all ThermoFischer Scientific). The products were sequenced on the ABI3130XL (Life Technologies Ltd., Paisley, UK and analysed with CLC Main Workbench (CLC bio, Aarhus, Denmark).

PCR enzyme	PCR primers	PCR reagents	PCR conditions	Comment	Result
Accuprime (ThermoFischer Scientific)	F1+R1	10x AccuPrime buffer II 1.25µl<br>Primer F 2µM 1.13µl<br>Primer R 2µM 1.13µl<br>Accuprime 2U/ul 0.25µl<br>dH_2O 7.75µl<br>DNA ≈50ng/ul 1.00µl	95ºC 2min + 95ºC 30s, 61ºC 30s , 69ºC 1min x 35 cycles + 72ºC 10 min + 4ºC →	PCR program with 69 ºC elongation temperature. Recommended from manufacturer for AT rich templates	Allele drop-out of 13AT allele
Accuprime (ThermoFischer Scientific)	F1+R1	10x AccuPrime buffer II 1.25µl<br>Primer F 2µM 1.13µl<br>Primer R 2µM 1.13µl<br>3 % DMSO 0.38µl<br>Accuprime 2U/ul 0,25µl<br>dH_2O 7.38µl<br>DNA ≈50ng/ul 1.00µl	95ºC 2min + 95ºC 30s, 61ºC 30s , 72ºC 1min x 35 cycles + 72ºC 10 min + 4ºC →	PCR reaction with 3% DMSO Recommended by the manufacturer to loosen up secondary structures	Allele drop-out of 13AT allele
Platinum SuperFi II PCR Master MIX (ThermoFischer Scientific)	F1+R1	2x Platinum SuperFi II PCR mastermix 6.25µl<br>Primer F 2µM 2.50µl<br>Primer R 2µM 2.50µl<br>dH_2O 0.25µl<br>DNA ≈50ng/ul 1.00µl	98ºC 30s + 98ºC 10s, 61ºC 10s , 72ºC 30s x 35 cycles + 72ºC 5 min + 4ºC →	PCR enzyme recommended by ThermoFischer Scientific. PCR set-up and program based on manufacture recommendations.	Allele drop-out of 13AT allele
Herculase II Fusion DNA pol (Agilent Technologies)	F1+R1	5x herculase II buffer 2.50µl<br>Primer F 2µM 1.13µl<br>Primer R 2µM 1.13µl<br>dNTP mix, 10mM each 0.31µl<br>dH_2O 3.31µl<br>Herculase II fusion DNA pol 0.13µl<br>DNA ≈50ng/ul 1.00µl	95ºC 2min + 95ºC 30s, 61ºC 30s , 72ºC 1min x 35 cycles + 72ºC 10 min + 4ºC →	PCR set-up and program based on manufacture recommendations.	Allele drop-out of 13AT allele

Q5 Hi Fidelity DNA pol (New England BioLabs)	F1+R1	5x Q5 Reaction buffer 2.50µl<br>Primer F 2µM 3.13µl<br>Primer R 2µM 3.13µl<br>dNTP, 10mM 0.25µl<br>dH_2O 2.38µl<br>Q5 HiFid DNA pol 0.13µl<br>DNA ≈50ng/ul 1.00µl	98ºC 30s + 98ºC 10s, 61ºC 30s , 72ºC 30s x 35 cycles + 72ºC 2 min + 4ºC →	PCR set-up and program based on manufacture recommendations.	Allele drop-out of 13AT allele
OneTaq Hot Start DNA polymerase (New England BioLabs)	F1+R1	5x OneTaq Std Rxn buffer 2.50µl<br>Primer F 2µM 1.13µl<br>Primer R 2µM 1.13µl<br>dNTP, 10mM 0.25µl<br>dH_2O 6.44µl<br>OneTaq Hotstart DNA pol 0.13µl<br>DNA ≈50ng/ul 1.00µl	94ºC 30s + 94ºC 30s, 61ºC 30s , 68ºC 1min x 35 cycles + 68ºC 5 min + 4ºC →	PCR set-up and program based on manufacture recommendations.	Both 8AT and 13AT alleles detected
Type iT Microsattelite PCR kit (QIAGEN)	F1+R1	2x Type-it PCR mix 6.25µl<br>Primer F 2µM 0.63µl<br>Primer R 2µM 0.63µl<br>dH_2O 3.00µl<br>DNA ≈50ng/ul 1.00µl	95ºC 5min + 95ºC 30s, 60ºC 90s, 72ºC 30sec x 28 cycles + 60ºC 30min + 4ºC →	Enzyme used at Telemark Hospital for expansion analysis.<br>PCR set-up and program based on manufacture recommendations.	Both 8AT and 13AT alleles detected
Advantage GC LA Polymerase (Takara)	E5F+E7R	2x GC Melt Buffer 12.50µl<br>dNTP-Mix (20mM each) 0.50µl<br>Primer F 1µM 1.00µl<br>Primer R 1µM 1.00µl<br>Nuclease free dH_2O 8.75µl<br>LA Polymerase Mix (5U/µl) 0.25µl<br>DNA ≈50ng/ul 1.00µl	94ºC 1min + 94ºC 30s, 63ºC 30s, 72ºC 1min x 30 cycles + 72ºC 5min + 4ºC →	PCR set-up and program based on manufacturer's recommendations	Both 8AT and 13AT alleles detected

Fragment Analysis (FLA)

Electrophoretic length separation of the PCR products was performed on 3130XL Genetic Analyzer (Life Technologies). Data were analyzed by GeneMarker v. 1.85 (SoftGenetics LLC).

**PCR enzyme**	**PCR primers**	**PCR reagents**		**PCR conditions**
Accuprime (ThermoFischer Scientific)	F1-FAM tagget+R1	10x AccuPrime buffer II<br>Primer F 2µM<br>Primer R 2µM<br>Accuprime 2U/ul<br>dH_2O<br>DNA ≈50ng/ul	1.25µl<br>1.13µl<br>1.13µl<br>0.25µl<br>7.75µl<br>1.00µl	95ºC 2min + 95ºC 30s, 61ºC 30s , 72ºC 1min x 35 cycles + 72ºC 10 min + 4ºC →
Type iT Microsattelite PCR kit (QIAGEN)	F1-FAM tagget+R1	2x Type-it PCR mix<br>Primer F 2µM<br>Primer R 2µM<br>dH_2O<br>DNA ≈50ng/ul	6.25µl<br>0.63µl<br>0.63µl<br>3.00µl<br>1.00µl	95ºC 5min + 95ºC 30s, 60ºC 90s, 72ºC 30sec x 28 cycles + 60ºC 30min + 4ºC →

Sequence traces for *MME:* c.467del

Individual with one short and one long AT-repeat

False homozygous deletion with primers outside AT-repeat

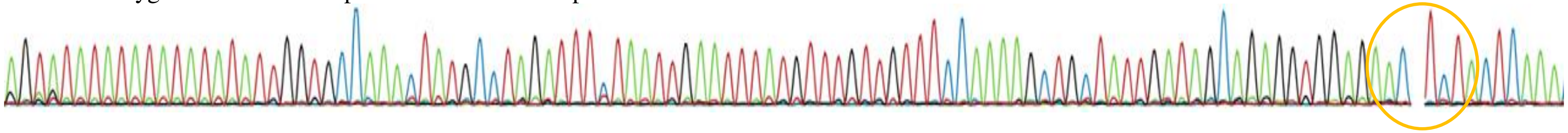

Correct heterozygous deletion with primers inside AT-repeat

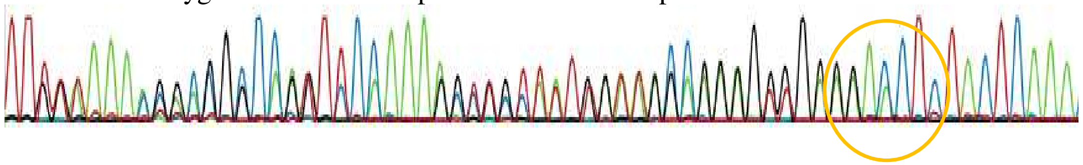

Individual with two short AT-repeats

Correct heterozygous deletion with primers outside AT-repeat

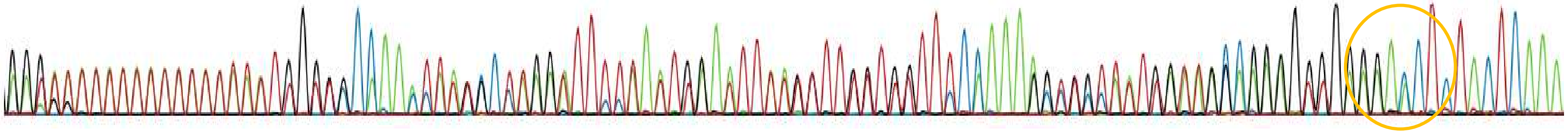

Correct heterozygous deletion with primers inside AT-repeat

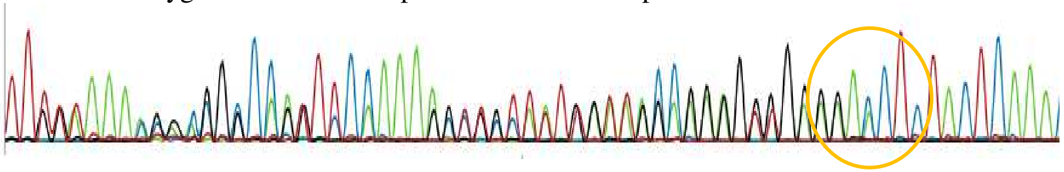

Sequence traces for *MME*: c.440-2A>C

Individual with one short and one long AT-repeat

False wildtype with primers outside AT-repeat

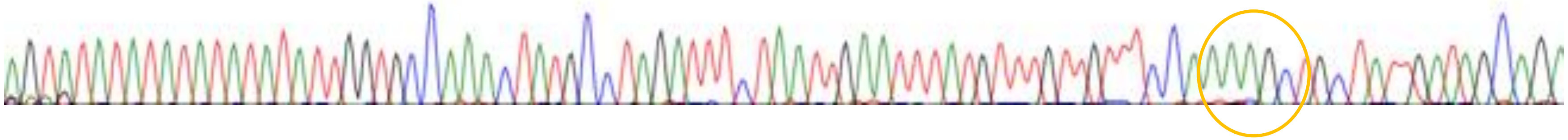

Correct heterozygous missense variant with primers inside AT-repeat

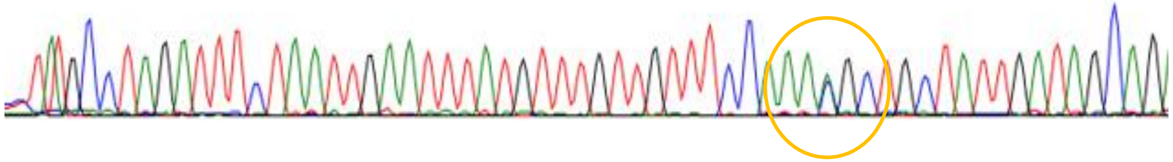

Individual with two long AT-repeats

Correct heterozygous with primers outside AT-repeat

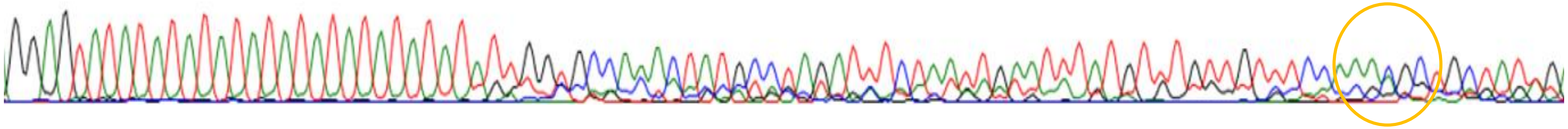

Correct heterozygous with primers inside AT-repeat

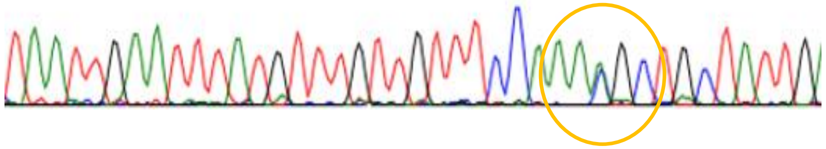

Supplement: Supplementary data [file jmedgenet-2021-108281supp001.pdf]
